# Knockdown of *Mct1* in the arcuate nucleus increases food-anticipatory activity in mice

**DOI:** 10.3389/fphys.2025.1642386

**Published:** 2025-09-26

**Authors:** Tomaz Martini, Urs Albrecht

**Affiliations:** ^1^ Institute of Biomedical Sciences, Faculty of Medicine, University of Maribor, Maribor, Slovenia; ^2^ Faculty of Science and Medicine, University of Fribourg, Fribourg, Switzerland

**Keywords:** daytime-restricted feeding, circadian, feeding, arcuate nucleus, AGRP, MCT1, monocarboxylate transporter, SLC16A1

## Abstract

Animals rely on internal time-keeping mechanisms to anticipate regular events such as feeding, allowing preemptive gene expression which enables timely physiological responses. A manifestation of anticipatory mechanisms is also a rise in body temperature and activity before a predictable mealtime. The activity, which resembles food seeking, depends on the communication between peripheral organs and the brain. The liver plays a central role by producing metabolic signals, including beta-hydroxybutyrate, which is released into the blood in anticipation of feeding. This release is controlled by the transporter MCT1, and its hepatic ablation in mice impairs food-anticipatory activity (FAA). However, in parallel, loss of MCT1 in the arcuate nucleus, a brain nucleus that orchestrates feeding behaviour, was implicated in increased food intake, creating a paradox. Here, we demonstrate that MCT1’s role in feeding behavior is tissue dependent, and that while hepatic and systemic disruption of *Mct1* impair FAA, arcuate nucleus *Mct1* knockdown increases FAA. This underscores the complexity of small molecule signalling in metabolism, of which MCT1 is merely a transporter, and whose actions are ligand, and hence context and tissue dependent.

## Introduction

Mammals are complex organisms capable of responding dynamically to the environment. Many of these physiological changes are repeated daily as part of the adaptation to life on Earth. To better anticipate these recurring changes and minimize the delay between sensing external cues and triggering a protein-mediated response—an inherently slow process—organisms have evolved anticipatory mechanisms driven by a genetically programmed molecular clock. This clock allows for gene expression before a recurring event, such as the animal’s first meal upon waking, ensuring that essential enzymes for nutrient processing are readily available. The molecular clock improves efficiency by coordinating biological processes, managing competition for shared substrates, and reducing the risk of toxic metabolite production.

A main role of the molecular clock is the anticipation of recurring feeding and fasting cycles in animals. To achieve a coordinated response to feeding, organs need to work in a synchronised manner, which is achieved by inter-organ communication via both innervation and humoral cues (Martini et al., 2023). One of the identified humoral cues involved in temporal coordination of feeding behaviour is beta-hydroxybutyrate (bOHB) of hepatic origin. Indeed, the production of bOHB in the liver is coordinated by the molecular clock, and in particular the clock gene *Per2*. Mice with a hepatic *Per2* deletion or perturbed hepatic bOHB export machinery have difficulties adapting to changes in their feeding schedule (Chavan et al., 2016; Martini et al., 2021a). More specifically, these otherwise nocturnal animals fail to exhibit a typical food-anticipatory phenotype under recurring daytime feeding. This phenotype is characterized by a rise in internal body temperature and increased activity—resembling food-seeking behaviour—in the hours before food is recurrently presented.

A key player in hepatic bOHB efflux is the monocarboxylate transporter 1 (*Mct1*/*Slc16a1*), a bidirectional transporter of short-chain fatty acids, ketone bodies and lactate (Halestrap and Wilson, 2012; Bergersen, 2015). As such, MCT1 plays an integral part in the convergence of circadian and metabolic signaling on the organismal level. Mice that lack *Per2* in the liver lose their normal rise in body temperature before feeding and their food-anticipatory activity (FAA). In contrast, hepatic *Mct1* knockouts (KOs) can still adjust their body temperature rhythms, but exhibit severely attenuated FAA under daytime-restricted feeding (dRF). Hence, a deletion of *Mct1* likely cuts the communication axis between the peripheral metabolic tissues and the brain. Indeed, even whole-body heterozygous *Mct1* mice, carrying a deletion of one *Mct1* allele, display markedly reduced FAA (Martini et al., 2021a).

The multiple substrates of MCT1 add complexity to its role in metabolic signalling. Under dRF, circulating bOHB increases around feeding time, whereas pyruvate and lactate remain unchanged. Hepatic *Mct1* KO abolishes the feeding-associated peak in bOHB under dRF, while leaving circulating pyruvate and lactate levels unaffected (Martini et al., 2021a). Hence, at the periphery, bOHB is likely a critical signaling molecule. Consistently, in mice unable to generate sufficient bOHB to sustain FAA, the anticipatory phenotype can be rescued by temporally programmed bOHB delivery via implanted minipumps (Chavan et al., 2016).

However, these findings—particularly those from whole-body *Mct1* heterozygous models—appear to contrast with reports that *Mct1* knockdown (KD) in tanycytes of the arcuate nucleus (ARC), a key metabolic brain region, increases food intake in rats (Elizondo-Vega et al., 2016). It is known that *Mct1* has a double faceted role. It handles ketone bodies in the periphery, which signal a fasting state (Martini et al., 2021a), but it is also instrumental in allowing astrocytes—and possibly other glia—to provide lactate to neurons as a source of energy (Pellerin, 2005), thereby signalling to neurons that energy supplies are plentiful. Exploring this dual role of *Mct1*—in both peripheral metabolic signaling and central energy supply—could provide critical insight into the basic regulation of feeding behavior.

While highly informative, previous state-of-the-art experiments describing ARC MCT1’s role in feeding did not account for circadian influences. This raises the question whether *Mct1* manipulation disrupts the temporal regulation of ARC function and attenuates dynamic physiological programmes, such as the shift from fasting in the mammalian rest phase to feeding. Indeed, recent findings indicate that even the permeability of the blood-brain barrier (BBB) between the ARC and the adjacent median eminence (ME)—intrinsically tied to tanycyte function—fluctuates throughout the day, allowing nutrients to enter the core of the ARC in a rhythmic manner (Rodríguez-Cortés et al., 2022). Given the dynamic physiological properties of the ARC, and to reconcile the apparent paradox—where MCT1 can either suppress food-seeking behavior or enhance energy intake depending on its specific localization—we analysed monocarboxylate transporter expression at the single-cell level in the ARC and conducted circadian behavioural experiments on the mice with ARC-specific *Mct1* KD.

## Results

**FIGURE 1:**
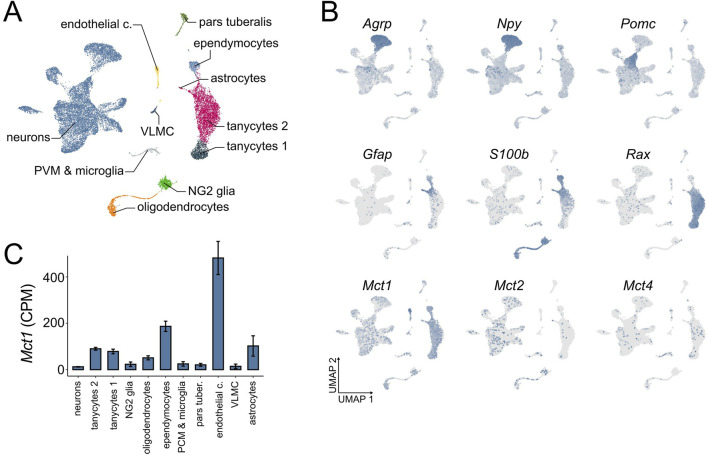
ARC scRNA-seq at ZT 2–4. **(A)** A UMAP dimensionality reduction plot showing the cell types of the ARC and ME area, independent of feeding condition in the dataset (n = 53). **(B)** Distribution of marker gene and monocarboxylate transporter expression across cell types of the ARC and ME, independent of feeding condition. Gray indicates no expression, while increasing blue intensity corresponds to higher relative expression levels (n = 53). **(C)** Expression of *Mct1* within different cell types of the ARC and surrounding tissue under *ad libitum* feeding with regular chow, expressed as raw counts per million (CPM), calculated for each cell within the cluster, and presented as mean cluster values +/- SE between cells.

To descriptively assess the expression patterns of monocarboxylate transporters in the ARC, we performed a focused re-analysis of ARC single-cell RNA sequencing (scRNA-seq) data (Campbell et al., 2017). This dataset revealed a diverse population of cell types within the ARC (Figure 1A). Among these, we observed that *Mct1* is broadly expressed across multiple cell types, with particularly high expression in endothelial cells, followed by ependymocytes, astrocytes and tanycytes (Figures 1B,C). These findings suggest that the role of *Mct1* in the ARC extends beyond its function in tanycytes.

Despite low baseline expression levels in neurons, the physiological role of neuronal *Mct1* cannot be fully dismissed as neurons comprise nearly two-thirds of the total cell population in the scRNA-seq dataset—consistent with neuronal proportions reported for the ARC in the Blue Brain Cell Atlas (Erö et al., 2018). Indeed, we have recently demonstrated that even lowly expressed membrane proteins can be physiologically highly relevant (Martini et al., 2024).

The ARC-ME scRNA-seq dataset comprises different feeding conditions in addition to *ad libitum* (AL) food availability on regular chow. Mice were also fed a low fat or high fat diet, or subjected to overnight fasting with or without subsequent refeeding (Supplementary Figure S1). In the original study, all mice were sacrificed between ZT 2 and 4 (2–4 h after lights-on; see Methods) to capture the transcriptomic effects of these nutritional perturbations. The consistent time-window of sacrifice minimises circadian variability across conditions. Moreover, the time of sacrifice coincides with the preprandial rise in body temperature and locomotor activity observed under the dRF protocol, making this dataset particularly well-suited for our study. Although the overnight fasting shares similarities with the fasting period under dRF, the key difference is the absence of a shift in the hepatic clock during the overnight fast. This makes it an ideal model for studying the acute, cell-specific responses to fasting in the absence of the broader temporal reprogramming that occurs under dRF. Indeed, mice need several days to adapt to the dRF regime. After overnight fasting, neuronal *Mct1* is upregulated, reaching almost 2-fold baseline expression levels, while the upregulation of *Mct1* upon overnight fasting in glia is non-significant, with the exception of ependymocytes, in which *Mct1* expression is downregulated (Supplementary Figure S1). These results suggest that, among the numerous ARC cell types, neurons dynamically regulate *Mct1* expression levels based on nutrient conditions.

**FIGURE 2:**
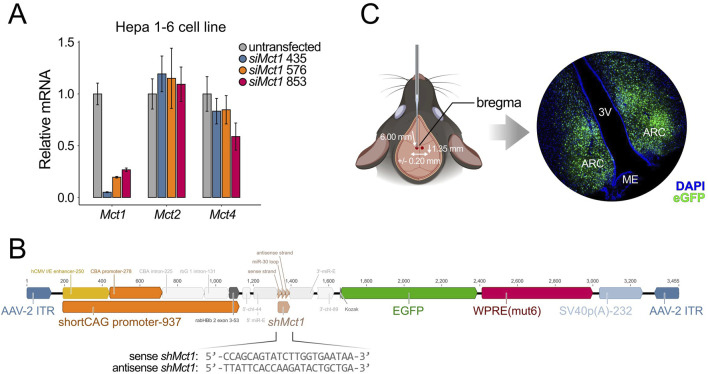
**(A)** *In vitro* siRNA assay on Hepa 1-6 cells, a hepatocellular carcinoma cell line. Cells were transfected with *siMct1*, and *Mct* mRNA assessed the following day. siRNA constructs against different target sequences of *Mct1* exons were tested for KD efficiency. Among the sequences tested, *siMct1* 435 showed an approx. 95% reduction of *Mct1* mRNA, with non-significant effects on *Mct2* and *Mct4* (n = 3, per condition). **(B)** The 435 siRNA sequence was transformed into an shRNA sequence by adding a hairpin. This sequence was printed and cloned into an AAV2 expression cassette, which was used for viral production. The virus was packed into AAV9 capsids. **(C)** The AAV9-*shMct1* virus was stereotactically injected bilaterally into the ARC, with coordinates of −1.35 mm anterior–posterior, +/-0.20 mm medio–lateral and −6.00 mm dorso–ventral, and a volume of 100 nL per hemisphere at 4.2 × 10^12^ vg/mL. The total volume was injected at an approximate rate of 20 nL per min. Since the expression vector contained an *eGfp* construct, the transduction could be monitored with microscopy on caudal brain sections. Control animals were injected with an equivalent virus, with a scramble sequence in place of *shMct1*.

To investigate the functional role of ARC *Mct1*, we set out to perform brain-region-specific viral-mediated *Mct1* KD and subject the animals to dRF. As a first step, we identified three candidate target sequences for *Mct1* silencing. We then designed and tested siRNA constructs against these targets *in vitro* for *Mct1* KD efficiency (Figure 2A). The *siMct1* constructs were designed to target *Mct1* sequences that were non-homologous to other monocarboxylate transporters. As a precautionary measure, we evaluated the effects of the three *siMct1* constructs on the two other monocarboxylate transporters with similar ligand specificity, *Mct2* (*Slc16a7*) and *Mct4* (*Slc16a3*). In the brain, *Mct2* is reported to be predominantly expressed in neurons, with a high affinity for lactate and pyruvate, and *Mct4* (*Slc16a3*) is reported to be primarily expressed in astrocytes, and important for astrocytic lactate efflux (Supplementary Table S1) (Halestrap and Wilson, 2012; Bergersen, 2015; Pérez-Escuredo et al., 2016). The most effective siRNA (435) was then selected for viral design and *in vivo* experiments to achieve targeted *Mct1* knockdown in the ARC while minimizing off-target effects. Viral design and production was carried out by the Viral Vector Facility (VVF) Zurich.

To enable stable expression, the selected construct was first modified by incorporating a loop sequence and extended into an shRNA construct (Fellmann et al., 2013), which was synthesized and cloned into an adeno-associated virus (AAV) vector plasmid provided by the VVF (Supplementary Figure S2A). With up to two mismatches, no target sequences of the shRNA sequence other than that of *Mct1* were identified in the mouse exome. Given the broad expression of *Mct1* across multiple ARC cell types, the expression cassette was designed to contain a shortened artificial cytomegalovirus enhancer/chicken beta-actin (short CAG) promoter, allowing for cell-type nonspecific expression. This plasmid was subsequently used for virus production, with viral packaging into AAV9 serotype capsids (Figure 2B). As a control, a viral expression vector with an identical expression cassette but with a nonsense shRNA sequence in place of the *shMct1* was used (scramble). These constructs were tested *in vivo* for their transduction and KD efficiency (Supplementary Figure S2B,C). The *shMct1* plasmid and viral vector have been deposited in the VVF Zurich Repository for broader accessibility, with control constructs readily available from the same resource (see Resource availability for details).

**FIGURE 3:**
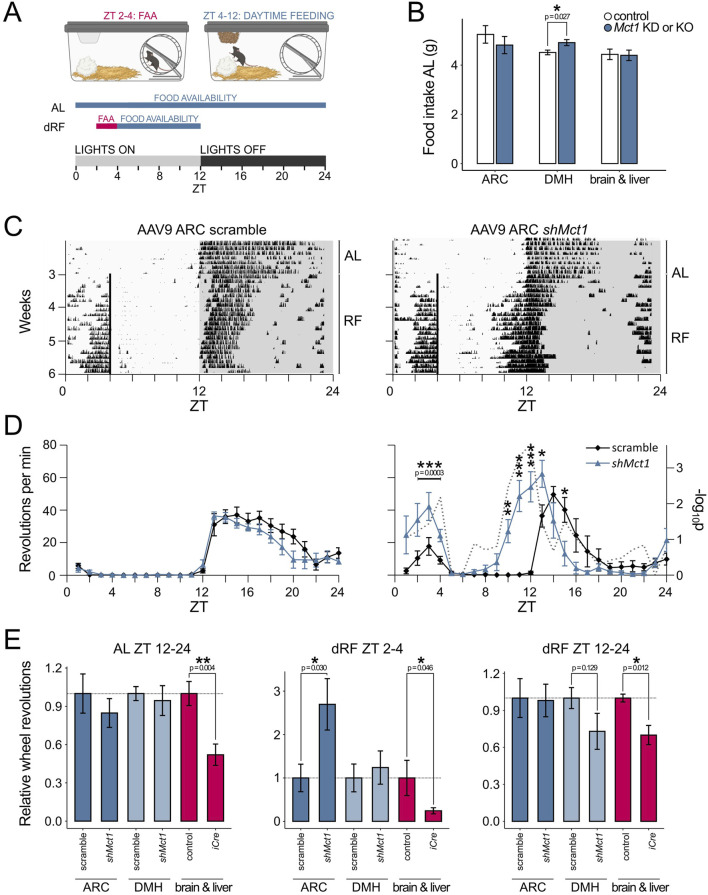
**(A)** Schematic representation of the daytime-restricted feeding (dRF) protocol. In circadian biology, the time of day is denoted by ZT (Zeitgeber time), where ZT 0 is the time when lights are switched on in an animal housing facility. In a 12 h light, 12 h darkness routine, the mice typically become active and begin eating at ZT 12, when lights are switched off. When food is provided recurrently at ZT 4 (dRF), the mice adapt to the new feeding schedule after approx. 3 days and start exhibiting food-anticipatory activity (FAA) in the hours before feeding. To account for lower caloric requirements under dRF and prevent daytime overeating, the food intake is limited to 80% in the first week and 70% in the following 2 weeks of the dRF protocol, calculated based on an individual mouse’s food intake during AL. **(B)** Average daily intake of regular chow over the 3 weeks AL period for ARC and DMH *Mct1* KD mice as well as of PHP.eB-injected animals and their respective controls. **(C)** Actograms showing the activity of a control and an ARC *Mct1* KD mouse. The lines represent individual days of the activity monitoring experiment, and each column’s height corresponds to the relative wheel-running activity at a respective time and day. Under AL, both the control and KD mice exhibit minimal activity during the light phase (ZT 0–12), but start running on the wheel immediately when the lights are switched off at ZT 12. Under dRF, the mice adapt to the feeding schedule after approx. 3 days. In the KD mouse, the FAA is increased, and the temporal pattern of nocturnal activity shows a phase advance. **(D)** Activity profiles of the last week of AL (left panel) and last week of dRF (right panel) show the average daily activity (+/- SE) of mice in the control (black line) and ARC *Mct1* KD groups (blue line). The ARC KD group shows altered behaviour under dRF, with increased FAA (n = 6, per group; 2-way ANOVA with Tukey’s *post hoc* test for ZT 2–4). The ARC KD also exhibits a phase advance of the onset of nocturnal activity; the gray dotted line depicts the -log_10_p (two-tailed t-test at each time-point; * - p < 0.05, ** - p < 0.01, *** - p < 0.001). **(E)** Relative wheel-running activity compared to controls within periods ZT 2–4 (FAA quantification) and ZT 12–24 (nocturnal activity quantification). The middle panel, showing quantified FAA, highlights the contrast between ARC-specific *Mct1* KD and systemic *Mct1* ablation. Activity was normalized to the respective littermate and scramble controls for the ARC (n = 6, per group) and DMH (n = 5-6, per group) KDs and brain-liver KO (PHP.eB; n = 4-5, per group; Student’s two-tailed t-test, error bars represent SE; * - p < 0.05, ** - p < 0.01, *** - p < 0.001).

Control mice and KDs were generated by stereotactic injections of the scramble or *shMct1*-expressing virus into the ARC under surgical depth anesthesia (Figure 2C), as previously described in detail (Martini et al., 2021b). Three weeks after surgery, allowing for a full recovery and expression of the viral constructs, the mice were placed into single cages equipped with a running wheel. The activity and food intake of mice was monitored for 3 weeks under *ad libitum* (AL) food availability, followed by 3 weeks of activity monitoring under dRF (Figure 3A). In contrast to the increased food intake reported in rats following tanycyte-specific *Mct1* KD (Elizondo-Vega et al., 2016), we observed no difference in food intake between ARC scramble and *Mct1* KD groups under AL (Figure 3B). However, following our injections, the viral marker was present predominantly in the core of the ARC, with limited spread to tanycytes (Figure 2C). Indeed, while we performed injections into the ARC parenchyma, the tanycyte manipulations were performed by injecting viruses into the third ventricle (Elizondo-Vega et al., 2016).

Additionally, no major differences between sham-injected and ARC *Mct1* KD mice were observed in activity patterns under AL feeding (Figures 3C,D). The mice were then subjected to a dRF schedule, in which their food access was limited to ZT 4–12 for 3 weeks, and caloric intake limited to 80% of their baseline AL intake in the 1^st^ week and 70% in the 2^nd^ and 3^rd^ week of dRF (Figure 3A). A detailed description of this paradigm is provided in our previous studies (Chavan et al., 2016; Martini et al., 2021a; Martini et al., 2018). Under dRF, ARC KD of *Mct1* resulted in increased locomotion before feeding-time compared to controls, without a phase shift (Figures 3C,D), indicating intact temporal programmes of daytime metabolic signalling. The increase in food-seeking behaviour may, however, reflect a higher food drive, although this interpretation requires further investigation.

ARC *Mct1* KD mice also initiated nocturnal activity before lights were switched off (Figures 3C,D), indicating a phase advance in their nocturnal activity pattern and a partial override of the normal light-induced masking of activity. This shift was accompanied by a modest increase of the nocturnal siesta—the period of reduced activity in the latter half of the dark phase.

To validate that the observed phenotypes were an ARC-specific manifestation, we also performed behavioral assessment of mice representing both an anatomical or systemic control. For the anatomical control, we injected an equivalent amount of the AAV9-*shMct1* virus into the dorsomedial hypothalamus (DMH), a key brain region involved in integrating circadian, metabolic and behavioral signals, with implications in FAA, and direct connections to the ARC (Tang et al., 2023; Acosta-Galvan et al., 2011). In our experiment, DMH *shMct1* mice showed a marginally higher food intake (Figure 3B), but equivalent locomotion compared to controls under AL. Under dRF, the DMH *shMct1* mice exhibited no difference in FAA compared to sham-injected controls, albeit with a small reduction in nocturnal activity under dRF (Figure 3E; Supplementary Figure S3). The anatomical control, with injections into a brain region that is located adjacent to the ARC, hence demonstrates that the increase in food-seeking-like behavior upon *Mct1* KD is an ARC-specific manifestation rather than a consequence of general hypothalamic *Mct1* manipulation.

To control for systemic effects of *Mct1* manipulation, we took advantage of the known tropism of the AAV-PHP.eB serotype, which has been shown to primarily transduce the brain and liver following systemic administration (Martini et al., 2021b; Kawabata et al., 2023; Mathiesen et al., 2020). To this end, we delivered an *iCre*-expressing AAV-PHP.eB virus via tail vein injections into adult *Mct1* floxed mice. In these mice, *Mct1* is targeted for deletion and is excised from *iCre*-expressing cells (Martini et al., 2021a). A modest dose of 10^11^ viral genomes (vg) per mouse was used, reducing hepatic *Mct1* mRNA by 60% and hypothalamic *Mct1* mRNA by 26%. Despite the modest reduction of hypothalamic *Mct1*, these mice exhibited reduced AL locomotion (Figure 3E, left panel), consistent with the observation from our previous constitutive neuronal or glial whole-brain *Mct1* KO mice (Martini et al., 2021a). Furthermore, despite the modest *Mct1* reductions, the viral-mediated KOs exhibited severely attenuated FAA under dRF (Figure 3E, middle panel), accompanied with reduced nocturnal activity (Figure 3E, right panel). The systemic control hence reduces FAA, consistent with hemizygous *Mct1* mice, and attenuates nocturnal activity patterns as in whole-brain *Mct1* KO models.

**FIGURE 4:**
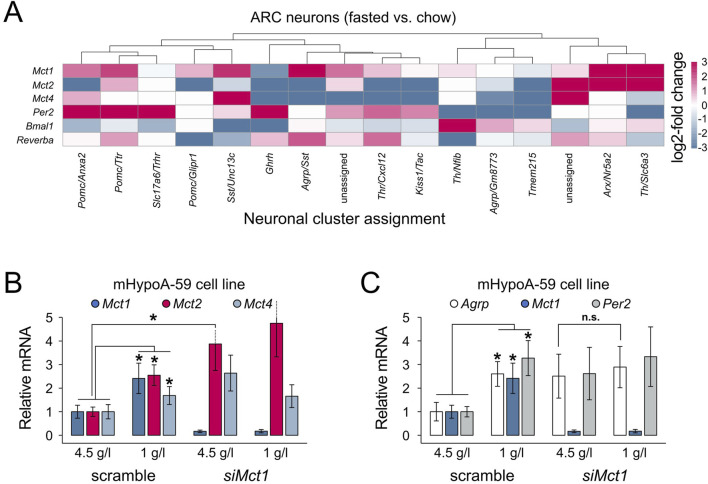
**(A)** Heatmap representing log_2_-fold changes in gene expression in the overnight fasted vs. regular chow condition, per neuronal cluster. *Mct1* shows highest upregulation in *Agrp*/*Sst*-, *Arx*/*Nr5a2*-and *Th*/*Slc6a3*-positive neurons. Non-ARC clusters, clusters without data-points and clusters with SE > relative mean expression are not shown. The log_2_-fold change was capped at −3 and 3. Two clusters from the original dataset could not be assigned to a defined neuronal subtype and are therefore indicated as “unassigned” (corresponding to clusters 33 and 34 in Campbell et al. (2017)). **(B,C)** Gene expression in mHypoA-59 cells under regular 4.5 g/L glucose medium conditions and under reduced (starvation) 1 g/L glucose. The cells were transfected with either a nonsense siRNA construct (scramble) or *siMct1*. **(B)** Reduced glucose levels increase *Mct1*, *2* and *4* expression, while *Mct1* KD leads to increased *Mct2* mRNA levels under regular medium conditions. **(C)** Both reduced medium glucose and *Mct1* KD lead to increased *Agrp* and *Per2* expression. Under *Mct1* KD, *Agrp* cannot be further upregulated under reduced glucose conditions compared to regular medium (pairwise comparisons using two-tailed unpaired Student's t-tests; n = 3; error bars represent SD; * - p < 0.05).

Together, we hypothesized that the higher FAA observed in ARC *Mct1* KD mice would likely be an output of neurons expressing the agouti-related peptide (*Agrp*), which in mammals increases food-seeking and feeding behaviour (Essner et al., 2017), either due to directly reduced neuronal *Mct1* or indirectly reduced *Mct1* in glia that provide nutrients to neurons. Indeed, at the single-cell level (Campbell et al., 2017), we subdivided the neurons based on their transcriptomic identity, and a subcluster of AGRP neurons, the *Agrp*/*Sst*-expressing neurons, showed a 4.2-fold expression of *Mct1* under fasting compared to regular chow (Figure 4A; Supplementary Figure S4). In addition, two smaller clusters of neurons, the *Arx*/*Nr5a2*- and dopaminergic *Th*/*Slc6a3*-positive cells, as well as one unidentified cluster, also showed higher mean levels of *Mct1* upon fasting. Conversely, *Agrp*/*Gm8773*- and *Pomc*-expressing subclusters exhibited a much smaller and non-significant response (Figure 4A; Supplementary Figure S4). Hence, these results suggest that a specific AGRP neuronal subtype dynamically upregulates *Mct1* as a response to energy deficits.

To further test this hypothesis in a controlled setting, we turned to an *in vitro* model. Using mHypoA-59 hypothalamic cells—which express ARC-characteristic neuropeptides, including *Agrp*, and retain key metabolic and nutrient-sensing properties of ARC neurons (Dhill et al., 2011)—we simulated intermittent starvation by culturing cells in reduced-glucose medium for 4 h, with or without siRNA-mediated *Mct1* KD. Consistent with the scRNA-seq data, this acute starvation led to increased expression of *Mct1* (Figure 4B), reinforcing the idea that *Mct1* is part of the fasting response in AGRP-positive hypothalamic neurons. The *in vitro* starvation also increased *Mct2* and *Mct4*, which was not the case in mice, where AGRP-neurons reduce *Mct2* and *Mct4* expression after an overnight fast (Figures 4A,B). *In vitro*, *Mct1* KD alone resulted in upregulation of *Mct2*, even in normal glucose, suggesting a compensatory mechanism.

The intermittent starvation of mHypoA-59 cells also led to a 2.5-fold expression of *Agrp* mRNA (Figure 4C), paralleling the increased *Agrp* expression in neuronal subtypes under fasting in our scRNA-seq analysis, and confirming that the mHypoA-59 cell line dynamically responds to metabolic state. However, in *Mct1* KD cells, *Agrp* expression was already elevated under basal conditions and did not further respond to starvation, indicating a dysregulation of nutrient-sensing responsiveness. Indeed, this could suggest that loss of *Mct1* may impair the ability of AGRP neurons to dynamically respond to metabolic cues—potentially contributing to the aberrant feeding behaviour observed in *Mct1* KD mice.

At first glance, it may appear somewhat paradoxical that starvation increases *Mct1* and *Agrp* in parallel, while *Mct1* KD also upregulates *Agrp*. However, these observations may reflect a shared underlying mechanism: in both cases, a perceived deficit in neuronal lactate availability—whether due to reduced systemic energy supply during fasting or impaired transport via MCT1—triggers a pro-feeding neuropeptide response (Figures 4B,C). In this model, MCT1 plays a dual role: its expression increases as part of the normal adaptive response to fasting, facilitating lactate uptake to support neuronal activity, but when absent or knocked down, the resulting energy stress alone may be sufficient to upregulate *Agrp*. Thus, MCT1 appears to function both as a mediator of nutrient sensing and as a component of the broader pro-feeding response.

Feeding fasting cycles are intrinsically connected to the molecular clock, but reduced neuronal lactate availability also elevates the NAD^+^/NADH ratio, enhancing the activity of SIRT1 (sirtuin 1), a master regulator of both feeding and the molecular clock PER2 protein’s degradation (Çakir et al., 2009; Bordone et al., 2007; Banks et al., 2008; Pfluger et al., 2008; Dietrich et al., 2010; Satoh et al., 2010; Levine et al., 2021). This prompted us to also evaluate clock gene expression in the data, especially focusing on *Per2*. Indeed, in the hypothalamus, the scRNA-seq data suggests altered molecular clock gene expression after overnight fasting, but in *Pomc*- and *Ghrh*-expressing neurons, and not *Agrp*-expressing neurons (Figure 4A). Conversely, in mHypoA-59 cells both *Mct1* KD and reduced glucose upregulate the expression of the molecular clock gene *Per2 in vitro* (Figure 4C). The observation that *Per2* mRNA is upregulated by both *siMct1* as well as reduced glucose conditions suggests a possible link between metabolic stress and circadian gene expression in hypothalamic neurons (Figure 4C). Indeed, it was previously shown that in the liver, *Per2* links glucose metabolism with circadian physiology (Zani et al., 2013).

Together, these findings underscore the importance of MCT1-mediated nutrient sensing in the ARC as a crucial component of anticipatory feeding behaviour. While peripheral bOHB may act as a systemic signal necessary for dRF adaptation, our data suggest that local lactate sensing within the ARC—modulated by MCT1—plays an active role in shaping neuronal responses to energy availability, possibly tipping the balance toward food seeking when nutrient uptake is impaired.

## Discussion

Given its strategic position dorsal to the median eminence and adjacent to a leaky blood-brain barrier—whose permeability fluctuates with the time of day (Rodríguez-Cortés et al., 2022)—the ARC is well-suited to function as a hub integrating peripheral and central signals (including projections from the hindbrain) with circadian programmes of energy homeostasis. This unique anatomical and functional placement suggests that ARC’s function can be manipulated by both local changes as well as organismal blood-borne nutrient signals. Indeed, the ARC manages both hunger and satiety signals, and it produces both orexigenic and anorexigenic outputs for feeding behaviour, fine tuning feeding behaviour to match the energetic needs of the organism in real time.

It is intriguing that ARC *Mct1* KD produces an effect opposite to that observed with reduced whole-body or ablated hepatic *Mct1* (Martini et al., 2021a), as well as with combined liver–brain partial *Mct1* deletion in adult animals, as demonstrated here. Indeed, a net result of organismal *Mct1* reduction is an attenuation of feeding behavior, whereas acting only on the ARC as a signal integrational hub drives increased food-seeking-like behavior. This contrast may reflect differences in the predominant ligand transported by MCT1 in each context: bOHB, produced in peripheral tissues such as the liver, may act as a systemic metabolic signal targeting specific brain regions (though the precise site of action remains unclear), whereas in the ARC, MCT1 primarily facilitates the neuronal uptake of lactate, a key energy substrate for neuronal activity. Indeed, we previously showed that ablating hepatic *Mct1* prevents the preprandial increase in blood bOHB, thereby eliminating this starvation-associated, pro-feeding signal (Martini et al., 2021a). Therefore, while increased preprandial circulatory bOHB is necessary for the adaptation to dRF, and hepatic MCT1 allows for the efflux of bOHB into the bloodstream, ARC *Mct1* KD likely prevents hungry neurons from receiving sufficient amounts of lactate from astrocytes, boosting food seeking.

While MCT1 is thought to predominantly transport lactate out of astrocytes, neuronal MCT1, which is dynamically regulated under starvation, could also help with neuronal lactate intake, as MCT1 is known to function as a bidirectional lactate transporter (Bola et al., 2014). This underscores the complexity of small molecule signalling in metabolism, of which MCT1 is merely a transporter, and whose actions are most likely ligand, and hence context and tissue dependent.

Indeed, the role of MCT1 in the body becomes even more complex in terms of dRF adaptation. Namely, our previous constitutive deletion of *Mct1* from *Gfap*- and *Nestin*-expressing cells only resulted in minor non-significant reductions of FAA, which were difficult to contextualize due to a generally lower activity of these animals. The scRNA-seq data here shows that in the ARC, only a small fraction of cells is *Gfap*-positive, while the *Nestin* driver itself shows metabolic effects (Harno et al., 2013), and the ARC is also a site of major AGRP neurogenesis (Yulyaningsih et al., 2017; Pierce and Xu, 2010; McNay et al., 2012), possibly circumventing developmental genetic manipulations.

Furthermore, it is interesting to note that ARC *Mct1* KD animals do not only exhibit increased FAA, but also a dramatic phase advance in their nocturnal activity, with locomotion when the mice would normally still be asleep. Notably, sleep disturbances have been associated with hyperphagic responses in both humans (Greer et al., 2013; Schmid et al., 2008) and rodents (Koban et al., 2008).

A limitation of our study is the observed modest spread of the AAV9 viral construct beyond the targeted ARC region (Figure 2C), which may have confounded region-specific effects. This spread is primarily seen as a dorsal reflux of the virus towards the DMH (Figure 2C), and was controlled for by an anatomical control injection. Although future studies could employ serotypes such as AAV8 or AAV5, which exhibit more restricted hypothalamic diffusion, the broader coverage of AAV9 ensured complete targeting of the ARC in the present study.

Moving forward, identifying the precise brain regions and cell types responsive to circulating bOHB, and dissecting how these interact with local nutrient-sensing circuits in the ARC, will be essential for a more complete understanding of how internal and external cues are integrated to govern rhythmic feeding behaviour. To overcome the limitations of constitutive *Cre*-*Lox* recombination—including developmental effects—genetic manipulations in adult animals, the use of AAV tracers, and immunohistochemical analyses to resolve the cellular expression patterns of monocarboxylate transporters will be key for advances in the field. Collectively, our findings suggest that MCT1 function is highly context- and location-dependent, exerting significant influence on feeding behavior.

## Methods

### Animals and housing

The housing and experimental procedures were performed according to Swiss and Slovenian legislation, and in accordance with the Declaration of Helsinki. All experiments were approved by the State Veterinarian of the Canton of Fribourg, Switzerland.

Before their introduction into the experimental setting, mice were group-housed in transparent plastic cages (267 mm long × 207 mm wide × 140 mm high; Techniplast Makrolon type 2 1264C001) with a stainless-steel wire lid (Techniplast 1264C116), with up to 4 animals per cage. Food and water were provided *ad libitum* (AL), and mice were kept in light- and soundproof ventilated chambers. All mice were entrained to a light-dark 12 : 12 cycle, and the time of day is expressed as zeitgeber time (ZT; ZT 0 represents lights on, ZT 12 denotes lights off). Two- to five-month-old males and females were used for the experiments unless otherwise stated, with the sexes equivalently distributed between groups.

The restricted feeding protocol, which was performed in custom-built wheel-running cages, has been described in detail elsewhere (Martini et al., 2021a; Martini et al., 2018). Briefly, mice were single-caged and the activity or temperature was recorded for 3 weeks under AL, and then for 3 weeks under dRF, with mice receiving 80% of their daily intake (measured during AL) in the first week and 70% in the following 2 weeks. Mice were fed at ZT4 (4 h after lights on) and food was removed at ZT 12 (just before lights off) in case any pellets remained. When entrained to RF, mice ate their portion of food during the first 3–4 h. During experiments, the temperature was maintained at 22 °C ± 3 °C. Activity was recorded based on revolutions of a running-wheel, which was mounted in the cage, and activity patterns were acquired and analyzed using the Actimetrics ClockLab software, Version 3.0 acquisition and 6.0.54 analysis. Schematics of experimental setups and animal procedures incorporate elements from BioRender.com.

Preprandial changes in activity profiles were compared using a 2-way ANOVA, with independent variables of ZT and genotype. Other time-points were compared between groups with the student’s two-tailed t-test. For single-point measurements, groups were compared with student’s two-tailed t-tests.

### siRNA assay

To test targets for KD, three siRNA constructs were designed and used at 40 µM working solution concentration: RNAi 435 (sense sequence (5′-3′): GCA GUA UCU UGG UGA AUA A; antisense sequence (5′-3′): UUA UUC ACC AAG AUA CUG C), RNAi 576 (sense sequence GGC UUG CUU UCA ACU UGA A), RNAi 853 (sense sequence (5′-3′): GGA AGC UGG AAA AUC UGA U; antisense sequence (5′-3′): AUC AGA UUU UCC AGC UUC C), all with a dTdT overhang at the 3′-end.

Cells were seeded on 6-well plates in 2.0 mL of antibiotic-free medium, with a targeted confluence of 30%–50% the following day, when transfection was performed. The protocol works best if the cells are not seeded densely. Before transfections, medium was exchanged to a volume of 1.75 mL per well. For 6 wells, reagents were prepared into two tubes, A and B. Tube A contained 875 µL Opti-MEM (Gibco) and 35 µL Lipofectamine RNAiMAX (Invitrogen) transfection reagent. Tube B contained 1,000 µL Opti-MEM and 1 µL siRNA at 40 µM working concentration. 875 μL of the solution from tube B was transferred into tube A, gently mixed by pipetting and incubated 15 min at RT. After incubation, 250 µL of the transfection mixture was added to each well dropwise. Cell lysis was performed after 24 h by removing the medium, washing the cells with PBS and adding the Macherey-Nagel LBP buffer from the Macherey–Nagel RNA Plus kit.

### mHypoA-59 starvation assay

The starvation-like conditions were simulated with a medium containing 1.0 g/L glucose (Sigma-Aldrich D5546). In both the 4.5 and 1.0 g/L glucose conditions the medium was supplemented with 5% FBS. After 4 h, the cells were lysed in Macherey-Nagel LBP buffer.

### Gene expression analysis

For gene expression analysis, the RNA was isolated using the Macherey–Nagel RNA Plus kit and reverse transcribed using the Invitrogen SuperScript II. qPCRs were performed using the RotorGene 6000, with the KAPA Probe or KAPA SYBR master mix reagent (Kapa Biosystems). The primer list was previously published (Martini et al., Frontiers in Physiology, 2021), while for *Agrp* the primers used were as follows: FW: 5′-GGC AGA AGC TTT GGC GGA GGT-3′, RV: 5′-AGC AGG ACT CGT GCA GCC TT-3′, probe: PR: 5′-FAM-CCG CGA GTC TCG TTC TCC GCG TCG C-BHQ-1-3’.

### Plasmid and virus design

The plasmid expressing shRNA was produced by the Viral Vector Facility (VVF) of the Neuroscience Center Zurich, University of Zurich and ETH Zurich. Briefly, the shRNA insert was constructed using the GeneArt Gene Synthesis and cloned into an ssAAV vector plasmid provided by the VVF. The plasmid was amplified and purified with anion exchange columns (Macherey-Nagel NucleoBond Xtra), characterized by Sanger DNA sequencing and restriction enzyme analysis, and supplied at 835 ng/μL. The KD plasmid contained a sense 5′-CCAGCAGTATCTTGGTGAATAA-3′ and antisense 5′-TTATTCACCAAGATACTGCTGA-3′ *shMct1* sequence. With co-transfection with helper plasmids, the plasmid was used to produce viruses, which were purified with ultracentrifugation and diafiltration. The virus was supplied at a titer of 4.2 × 10^12^ vg/mL.

Equivalently, a virus with the same regulatory elements and fluorescent marker, but expressing a random or nonsense (scramble) construct instead of the *shMct1* was designed and generated by the VVF (VVF repository number v344-9), and supplied at a titer of 6.7 × 10^12^ vg/mL.

### Virus injections

The viral injections into WT mice were performed as previously described in detail (Martini et al., 2021b). Briefly, mice were anesthetized with 80 mg per kg ketamine and 0.30 mg per kg medetomidine. Before surgical procedures, the depth of anesthesia was checked by an absence of a reflex when pinching the skin between the toes of the mouse 5–10 min after application of the anesthetic. In case of a reflex, a quarter of the initial dose of the anesthetic was additionally administered. Mice were then placed into a stereotactic frame, onto a heating pad, and their eyes were protected by the administration of a hydrogel. The head was disinfected, and a 1.5 cm long incision made centrally to the skin. The skull sutures were visualized with 3% H_2_0_2_, administered carefully not to damage the skin. Based on anterior-posterior and medio-lateral coordinates, a dental drill was used to make holes into the skull at appropriate positions. Stereotactic injections were performed with a pulled glass pipette, which was aligned to the bregma. The *shMct1* and scramble virus were injected bilaterally into the ARC with coordinates, relative to the bregma, of −1.35 mm anterior–posterior, +/-0.20 mm medio-lateral and −6.00 mm dorso-ventral, and a volume of the virus of 100 nL per hemisphere. For DMH injections, 100 nL of the virus was injected per hemisphere with coordinates of −1.40 mm anterior–posterior, +/-0.25 mm medio-lateral and −5.30 mm dorso-ventral. The anesthesia was reversed by atipamezole in 5 times the dose of medetomidine. Surgical procedures were followed by administration of carprofen at 10 mg per kg and careful postsurgical monitoring of the animals.

For intravenous viral delivery, mice were sedated and chemically restrained with an intraperitoneal injection of 40 mg per kg body mass ketamine and 0.15 mg per kg medetomidine in saline and placed on a heating pad, while hydrogel was applied to their eyes. We injected 10^11^ viral genomes (VVF repository number v25-PHP.eB) per mouse in a total volume of 200 μL PBS via the lateral tail vein. The sedation was reversed with atipamezole in 5 times the dose of medetomidine.

## Resource availability

The *shMct1* plasmid and viral vector used in this study have been deposited in the repository of the Viral Vector Facility Zurich and are available for distribution to researchers upon request (VVF Accession Number v1195). The construct can also be packaged into alternative viral capsids. Researchers using these resources are requested to cite this publication.

## Data Availability

The raw data supporting the conclusions of this article will be made available by the authors, without undue reservation.
