# Supplementary material for: Knockdown of Mct1 in the arcuate nucleus increases food-anticipatory activity in mice
Source: Front Physiol. 2025 Sep 26;16:1642386. doi: 10.3389/fphys.2025.1642386 (PMC12512238; doi:10.3389/fphys.2025.1642386)
Supplement: Supplementary file 1 [file DataSheet1.pdf]

Knockdown of *Mct1* in the arcuate nucleus increases food-anticipatory activity – Supplementary Information

Supplementary Tables

Supplementary Table 1: ***Cell-type distribution and substrate specificity of monocarboxylate transporters.*** *Mct1 (Slc16a1), Mct2 (Slc16a7) and Mct4 (Slc16a3) are broadly expressed across mammalian tissues. Within the CNS, Mct1 and Mct4 exhibit higher levels in glia, while Mct2 is considered a neuronal transporter* [4,5].

	Substrates	Directionality	CNS cell-type specificity
MCT1	lactate, pyruvate, bOHB, acetoacetate, short-chain fatty acids	bidirectional H^+-coupled transporter	astrocytes, oligodendrocytes, endothelial cells of BBB
MCT2	lactate, pyruvate, bOHB, acetoacetate<br><br>higher affinity for lactate and pyruvate than MCT1	bidirectional H^+-coupled transporter	neurons (especially excitatory neurons)
MCT4	lactate (low affinity, high capacity), pyruvate (low affinity, minor role)	H^+-coupled transporter, predominantly for efflux<br><br>(specialized for lactate export)	astrocytes

Supplementary Figures

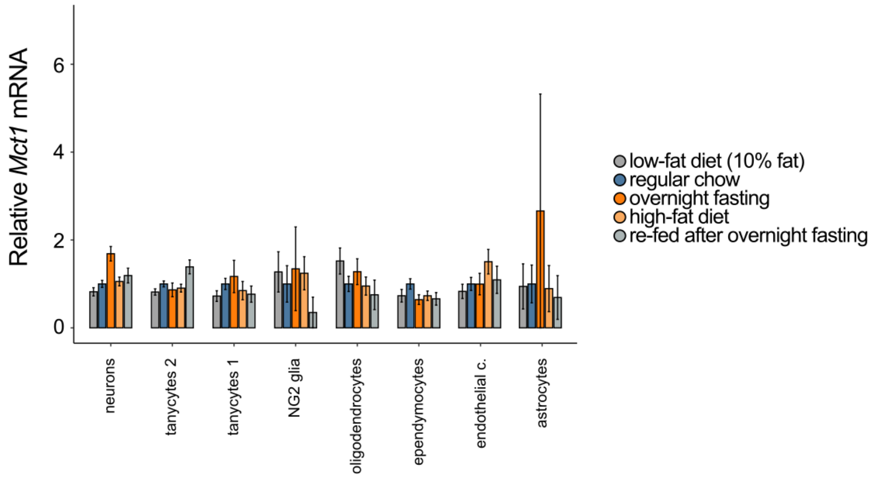

Supplementary Figure 1: Mct1 expression in ARC-ME cells under different feeding conditions. In neurons, overnight fasting increases Mct1 mRNA levels.

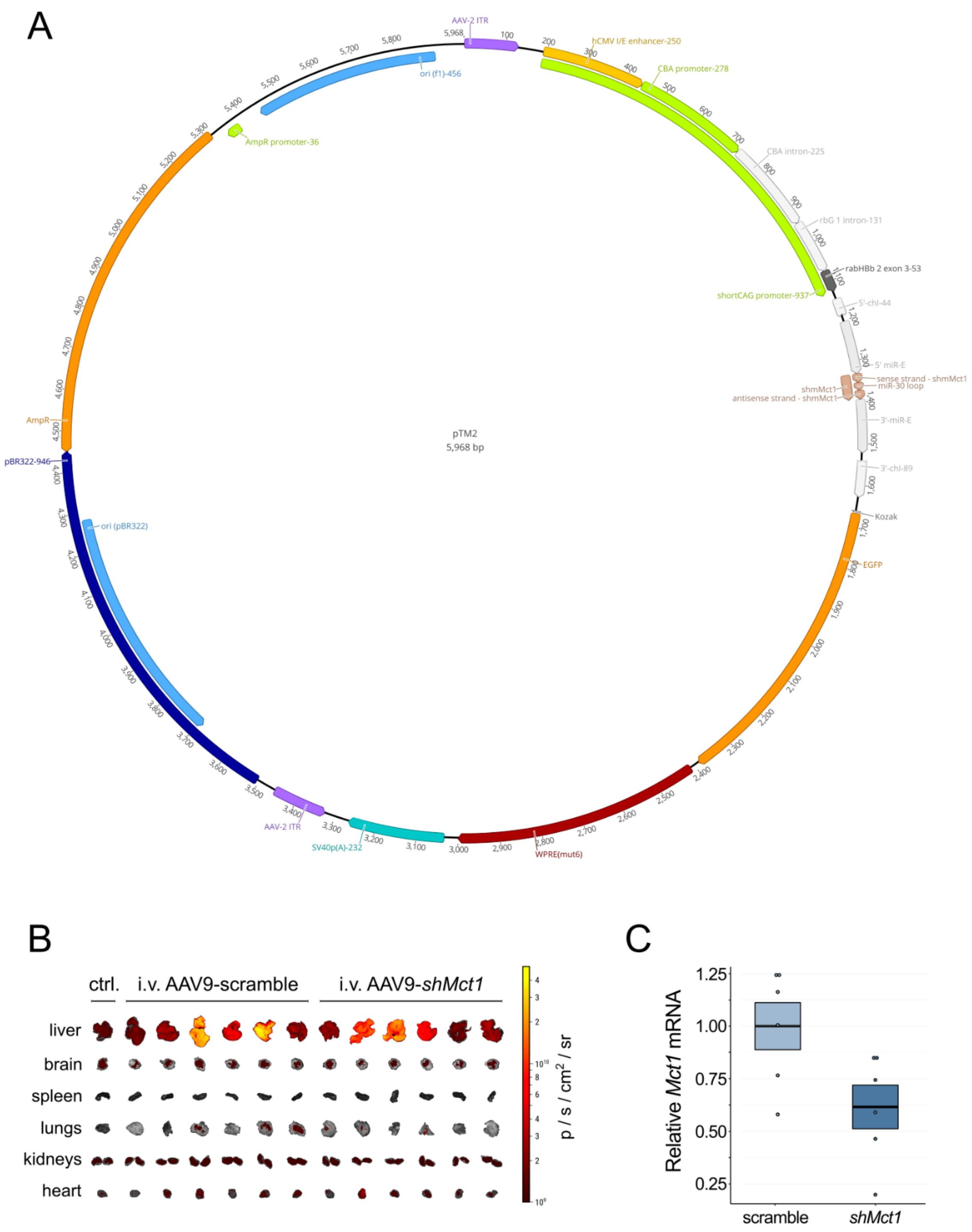

Supplementary Figure 2: (A) Plasmid map of pTM2 (5,968 bp). The plasmid expresses shMct1, provides resistance to ampicillin for bacterial selection and expresses the reporter eGfp (enhanced green fluorescent protein). (B-C) Validation of the AAV9-shMct1 construct in vivo. (B) Following tail vein injections at a titer of 10^{11} viral genomes per mouse, the virus primarily transduces the liver, based on the co-expressed enhanced Gfp. (C) Compared to the scramble construct, the modest viral titer reduces the hepatic Mct1 levels by one third.

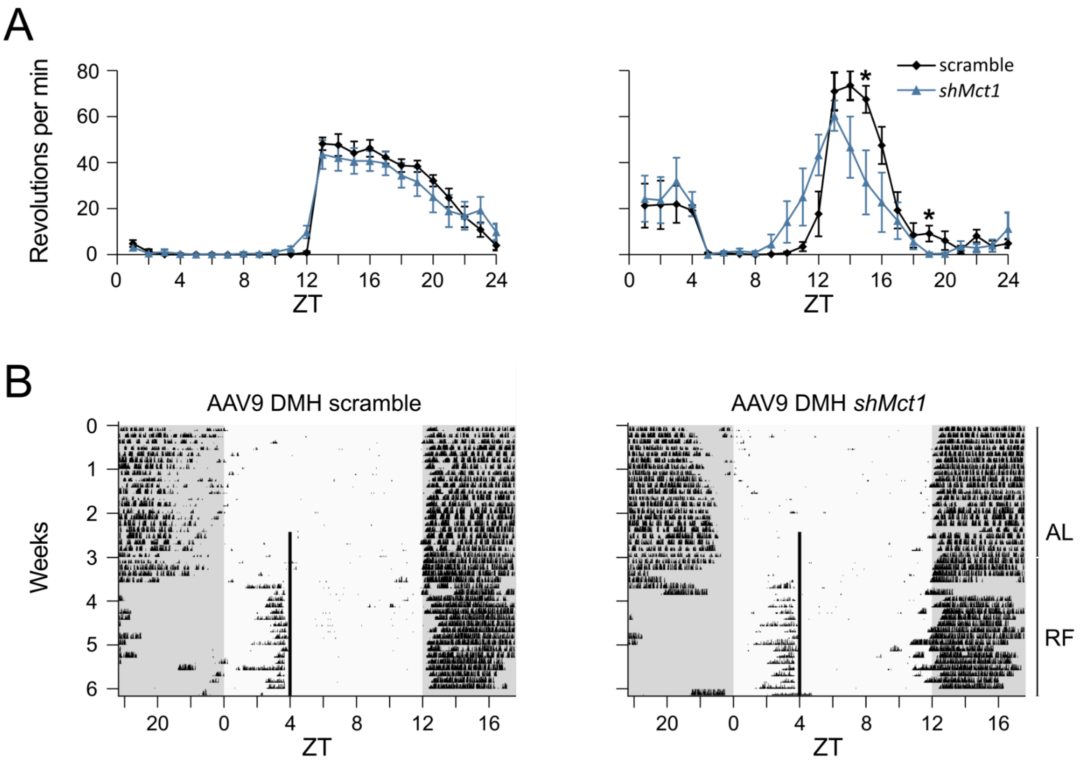

Supplementary Figure 3: Wheel-running activity of DMH-injected mice. (A) Activity profiles of the last week of AL (left panel) and last week of dRF (right panel) show the average daily activity (+/- SE) of mice in the control (black line) and ARC Mct1 KD groups (blue line). (B) Actograms show the activity of a control and a DMH Mct1 KD mouse. The lines represent individual days of the activity monitoring experiment, and each column's height corresponds to the relative wheel-running activity at a respective time and day. Under AL, both the control and KD mice exhibit minimal activity during the light phase (ZT 0-12), but start running on the wheel immediately when the lights are switched off at ZT 12. Under dRF, the mice adapt to the feeding schedule after approx. 3 days. In the KD mouse, the FAA appears comparable to the control, and the temporal pattern of nocturnal activity of the KD mouse is also aligned with the scramble-injected animal.

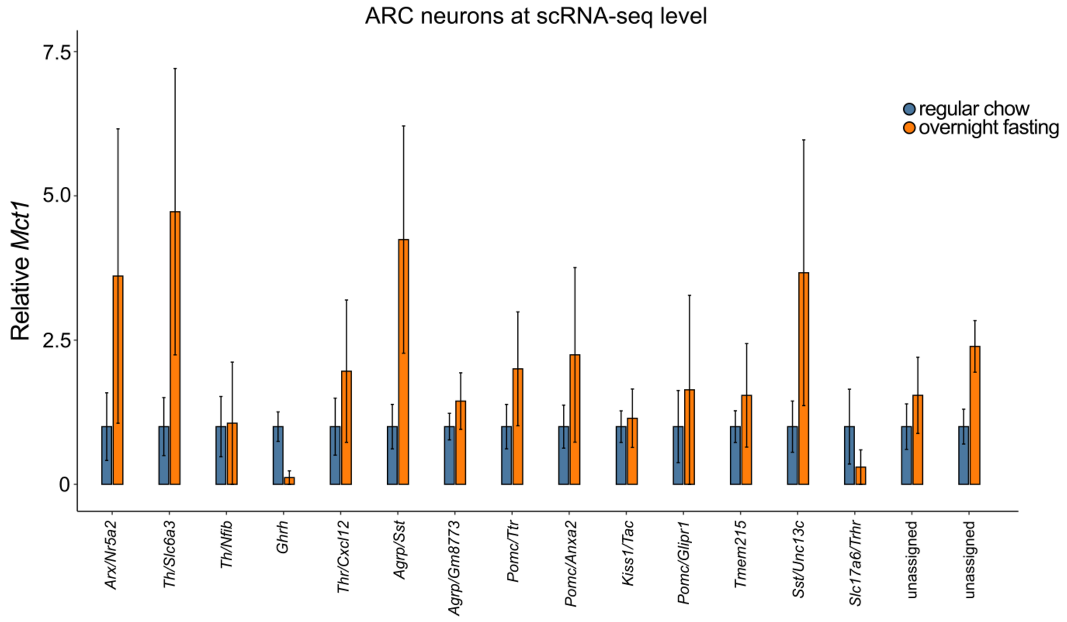

Supplementary Figure 4: Mct1 expression under regular chow has been normalised to 1 for each neuronal subcluster for easier visualization of relative changes in gene expression after the overnight fast. The overnight fast increases relative Mct1 levels only in a subset of ARC neurons, including Agrp/Sst-expressing neurons.
